# Supplementary material for: MetaRibo-Seq measures translation in microbiomes
Source: Nat Commun. 2020 Jun 29;11:3268. doi: 10.1038/s41467-020-17081-z (PMC7324362; doi:10.1038/s41467-020-17081-z)
Supplement: Supplementary file 10 — Supplementary Data 7 [file 41467_2020_17081_MOESM10_ESM.zip › File2/Confidence_VeryHigh_Taxonomy/148158_out.krona.html]

Javascript must be enabled to view this page.

members
magnitude
magnitudeUnassigned
count
unassigned
taxon
rank

148158\_out

4

2
4
superkingdom

phylum
1
201174

class
1
1760

85007
order
1

1
family
1653

1716
1
genus

43768

SRS017814\_contig\_number\_9291
1
species

1
phylum
976

class
1
200643

1
order
171549

family
1
171550

239759
genus
1

1896969

SRS018817\_contig\_number\_contig-100\_9035.9035
species
1

phylum
2
1239

class
1
186801

order
1
186802

541000
1
family

1
species

SRS104400\_contig\_number\_contig-100\_8003.262330
39492

1
class
91061

186826
order
1

family
1
1300

1301
genus
1


SRS144512\_contig\_number\_14361
1
species
1579343
